# The modified apron (Akil) incision: a technical modification to prevent subcutaneous emphysema in partial vertical and supracricoid laryngectomy

**DOI:** 10.1007/s00405-026-10186-z

**Published:** 2026-05-05

**Authors:** Ferit Akıl, Ahmet Çelik

**Affiliations:** 1 Memorial Diyarbakır Hospital, Uskudar University, Diyarbakır, Turkey; 2 Faculty of Medicine, Dicle University, Diyarbakır, 21400 Turkey

**Keywords:** Subcutaneous emphysema, Partial laryngectomy, Modified surgical technique

## Abstract

**Objective:**

To describe a modified apron (Akil) incision technique designed to prevent postoperative subcutaneous emphysema in patients undergoing open partial laryngectomy by maintaining an anatomical barrier between the laryngectomy field and the tracheostomy site.

**Methods:**

This study presents a technical modification applied in patients undergoing open partial laryngectomy, including supracricoid partial laryngectomy and partial verticallaryngectomy. The incision begins bilaterally at the mastoid tips, extends inferiorly along the anterior border of the sternocleidomastoid muscle, and terminatesapproximately 2 cm above the sternal notch. Unlike the conventional apron incision, an intact full-thickness skin bridge (approximately 2–2.5 cm in height) is preservedwithout undermining. All laryngeal framework procedures are performed superior to this bridge, while the tracheostomy is created inferiorly, preventing directcommunication between the two surgical fields.

**Results:**

A total of 23 patients were included. None developed clinically significant subcutaneous emphysema requiring intervention. Minor complications included one wound infection, one seroma managed with drainage and compression, and one case of partial wound dehiscence requiring revision. No complications were directly attributable to the incision design.

**Conclusion:**

The modified Akil incision provides a simple and effective anatomical barrier that may reduce the risk of postoperative subcutaneous emphysema following open partial laryngectomy. This technique is easily reproducible and may improve postoperative outcomes without increasing complication rates.

## Introduction

Subcutaneous emphysema following partial laryngectomy is typically caused by air leakage from the tracheostomy site or incomplete mucosal closure, particularly under conditions of coughing or positive-pressure ventilation. Although often self-limited, progressive SE may result in pneumomediastinum, respiratory compromise, or prolonged hospitalization [1–3].

The Modified Akil Incision was developed to create a **mechanical barrier** between the tracheostomy site and the cervical surgical field, thereby preventing air dissection into subcutaneous tissues. This manuscript describes the technical details of the procedure.

## Surgical technique

### Preoperative marking

The incision begins bilaterally at the mastoid tips and extends inferiorly along the anterior border of the sternocleidomastoid muscle. It terminates approximately **2 cm above the sternal notch**. 

**Fig. 1:**
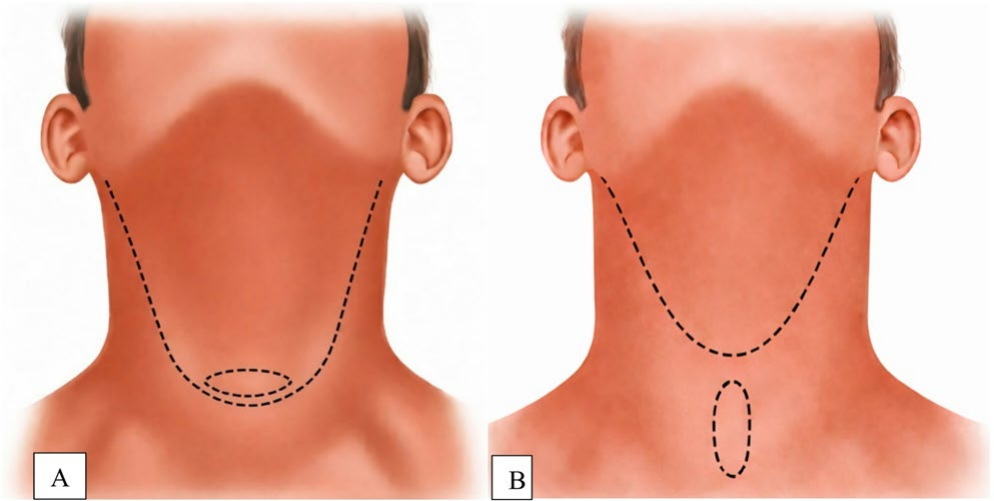
Schematic comparison of the conventional apron incision and the modified Apron (Akil) incision demonstrating the relationship between the tracheotomy site and the flap elevation field

Unlike the conventional apron incision, the inferior segments are not directly connected to the tracheostomy site. Instead, an inverted U-shaped superior flap is created while maintaining an intact intervening skin segment (Fig. 1).

(A) In the conventional apron incision, the tracheotomy site lies within the subplatysmal flap elevation area, creating a potential anatomical pathway for air tracking from the tracheostomy site into the dissected cervical soft tissues, thereby increasing the risk of postoperative subcutaneous emphysema.

(B) In the modified Apron (Akil) incision, the tracheotomy site is anatomically separated from the subplatysmal flap dissection field. This spatial separation disrupts the potential air dissemination pathway and reduces the likelihood of postoperative subcutaneous emphysema.

### Creation of the skin bridge

The key modification is preservation of a **2.0–2**,**5 cm full-thickness skin bridge** between:

- The inferior edge of the superior apron flap.
- The superior border of the tracheostomy incision.

### Technical principles

- The bridge includes skin and subcutaneous tissue.
- It is **not thinned or undermined**.
- Excessive cautery at the edges is avoided.
- Subdermal plexus integrity is preserved.

**Fig. 2:**
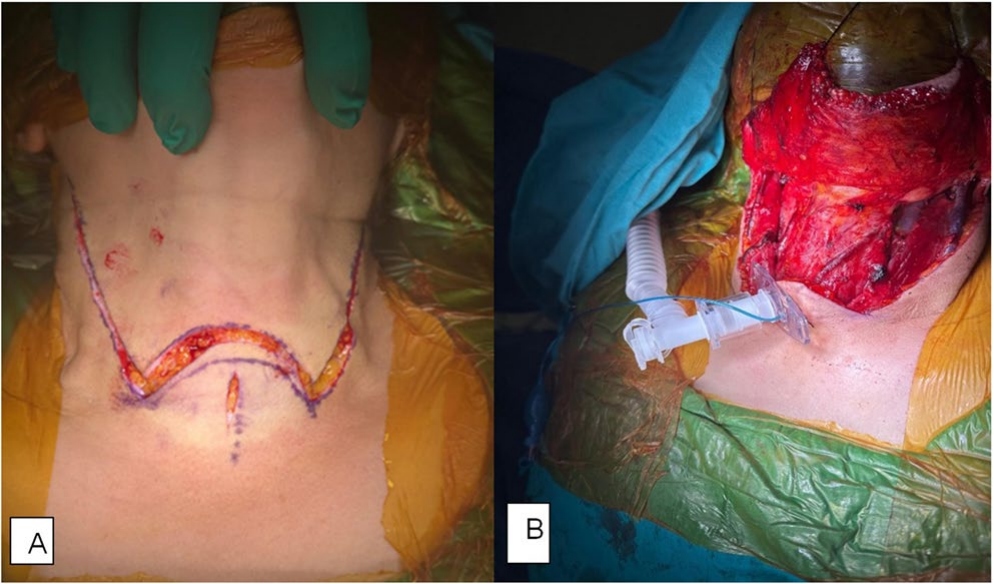
Intraoperative photographs. (**A**) Surgical field planning with marked incision lines and skin incision. (**B**) Operative field following flap elevation

This intact segment acts as a physical barrier preventing communication between the tracheostomy tract and the laryngectomy bed (Fig. 2). 

### Flap elevation

The superior apron flap is elevated in the standard subplatysmal plane.

No dissection is performed beneath the skin bridge.

**Fig. 3:**
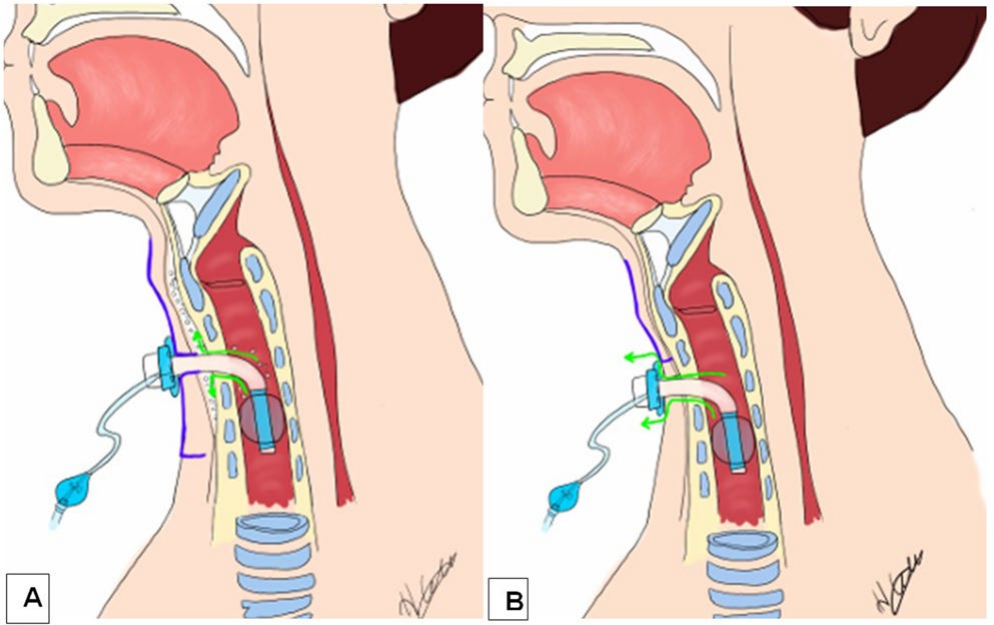
Lateral schematic comparison between the conventional apron incision and the modified Apron (Akil) incision

The bridge remains vascularized by lateral perforators and anterior cervical subdermal circulation (Fig. 3).

(A) Conventional apron incision. The blue line delineates the subplatysmal flap plane. The green arrows demonstrate the potential air dissemination pathway from the tracheostomy site into the flap elevation field, creating direct communication between the tracheostomy opening and the dissected cervical soft tissues, thereby increasing the risk of postoperative subcutaneous emphysema.

(B) Modified Apron (Akil) incision. The blue line delineates the subplatysmal flap plane, which is anatomically separated from the tracheostomy site. The green arrows illustrate the restricted air pathway. This spatial separation functions as a mechanical barrier, interrupting the potential communication between the tracheostomy site and the flap dissection plane and reducing the likelihood of postoperative subcutaneous emphysema.

### Tracheostomy formation

The tracheostomy is created through a separate inferior incision.

As a result:

- The laryngectomy field and tracheostomy site remain surgically isolated.
- Positive-pressure air cannot track superiorly into dissected cervical tissues.

### Exposure considerations

The preserved skin bridge does **not interfere** with:

- Thyroid cartilage exposure.
- Cricothyroid membrane access.
- Supracricoid reconstruction.

All laryngeal framework work is performed superior to the bridge.

### Patient selection

The technique is applicable in most open partial laryngectomy cases, including:

- Supraglottic laryngectomy.
- Vertical partial laryngectomy.
- Supracricoid partial laryngectomy (SCPL).

### Anatomical limitations

In patients with:

- Very short neck.
- Severe obesity.
- Extensive prior neck surgery.
- Radiation-induced fibrosis.

Maintaining a 2–2,5 cm bridge may be technically challenging. In such cases, meticulous preoperative planning is required.

### Postoperative management

Our postoperative protocol includes:

- Low-pressure cuff inflation immediately postoperatively.
- Early cuff pressure optimization.
- Compression dressing for 24–48 h.
- Active cough control.

Although cuff management alone does not prevent SE, combined with the skin bridge barrier it may reduce air leakage.

### Clinical experience

Between 2023 and 2025, the Modified Akil Incision was used in 23 consecutive patients undergoing open partial laryngectomy.

No clinically detectable or progressive subcutaneous emphysema occurred (Table 1).

Minor complications included:

- One wound infection (resolved with antibiotics).
- One seroma (US-guided drainage).
- One wound dehiscence (resutured).

No bridge necrosis was observed.

**Table 1:** Demographic and clinical characteristics of the patients

Characteristics	*n* (%)
Male	21 (%91,3)
Female	2 (%8,7)
Mean age (years)	62,7 +/-8,4
Chronic disease	11 (%47,8)
Smoking (> 10 years, ≥ 20 cigarettes/day)	23 (%100)
Alcohol use (> 5 years)	4 (%17,4)
Stage I	7 (%30,4)
Stage II	14 (%60,9)
Stage III	2 (%8,7)
Glottic	11 (%47,8)
Supraglottic	12 (%52,2)
Neck dissection	18 (%78,3)
Preoperative radiotherapy	3 (%13)
Albumin < 3,5 g/dL	1 (%4,3)

### Comparison with literature

According to the literature, the incidence of subcutaneous emphysema ranges between 3% and 12%, and this rate is particularly higher in SCPL-CHP procedures. A structured prevention protocol including layered closure, intraoperative Valsalva testing, negative-pressure drainage, and compressive dressings may significantly reduce the development of subcutaneous emphysema.

In a study by Lewin et al., subcutaneous emphysema was reported in approximately 26% of patients following supracricoid partial laryngectomy (SCPL), and it was identified as one of the most common postoperative complications [4].

In a study by Shi et al. evaluating 433 patients who underwent total or partial laryngectomy, the 30-day early postoperative complication rate in the partial laryngectomy group was reported as 19.4%, with a subcutaneous emphysema rate of 12.0% (*n* = 33) in this cohort [5].

Brumund et al. reported that among 270 patients operated on for laryngeal squamous cell carcinoma over a 25-year period, 49 patients (18.1%) developed significant postoperative surgical complications. The most common major complication was wound infection (19 patients; 7%), followed by seroma and major subcutaneous emphysema. However, the exact incidence of subcutaneous emphysema was not specified in this study [6].

In contrast, no clinically significant SE was observed in our cohort, suggesting that mechanical separation of surgical fields may reduce both incidence and severity.

### Surgical pearls

- Maintain a minimum **2 cm bridge width**.
- Avoid undermining beneath the bridge.
- Preserve subdermal vascular plexus.
- Prevent excessive tension in short-neck patients.
- Combine with airtight layered closure.

## Conclusion

Massive subcutaneous emphysema may lead to compartment syndrome, restriction of thoracic wall expansion, tracheal compression, and tissue necrosis. If left untreated, respiratory and cardiovascular compromise may develop [7].

Cases of severe respiratory distress caused by massive subcutaneous emphysema requiring emergency tracheotomy and surgical intervention have been reported in the literature [8].

The Modified Akil Incision is a simple, reproducible, and anatomically sound modification of the traditional apron incision. By creating a full-thickness skin bridge between the tracheostomy and the laryngectomy field, postoperative subcutaneous emphysema may be significantly reduced. The technique adds no operative time and does not compromise exposure.
